# Clinical Implications of Rabphillin-3A-Like Gene Alterations in Breast Cancer

**DOI:** 10.1371/journal.pone.0129216

**Published:** 2015-06-12

**Authors:** Balananda-Dhurjati Kumar Putcha, Xu Jia, Venkat Rao Katkoori, Chura Salih, Chandrakumar Shanmugam, Trafina Jadhav, Liselle C. Bovell, Michael P. Behring, Tom Callens, Ludwine Messiaen, Sejong Bae, William E. Grizzle, Karan P. Singh, Upender Manne

**Affiliations:** 1 Department of Pathology, University of Alabama at Birmingham, Birmingham, Alabama, United States of America; 2 Department of Epidemiology, University of Alabama at Birmingham, Birmingham, Alabama, United States of America; 3 Department of Genetics, University of Alabama at Birmingham, Birmingham, Alabama, United States of America; 4 Department of Medicine, University of Alabama at Birmingham, Birmingham, Alabama, United States of America; 5 Comprehensive Cancer Center, University of Alabama at Birmingham, Birmingham, Alabama, United States of America; Howard University, UNITED STATES

## Abstract

For the rabphillin-3A-like (*RPH3AL*) gene, a putative tumor suppressor, the clinical significance of genetic alterations in breast cancers was evaluated. DNA and RNA were extracted from formalin-fixed, paraffin-embedded (FFPE) cancers and matching normal tissues. DNA samples were assessed for loss of heterozygosity (LOH) at the *17p13*.*3* locus of *RPH3AL* and the *17p13*.*1* locus of the tumor suppressor, *TP53*. *RPH3AL* was sequenced, and single nucleotide polymorphisms (SNPs) were genotyped. RNA samples were evaluated for expression of *RPH3AL*, and FFPE tissues were profiled for its phenotypic expression. Alterations in *RPH3AL* were correlated with clinicopathological features, LOH of *TP53*, and patient survival. Of 121 cancers, 80 had LOH at one of the *RPH3AL* locus. LOH of *RHP3AL* was associated with nodal metastasis, advanced stage, large tumor size, and poor survival. Although ~50% were positive for LOH at the *RPH3AL* and *TP53* loci, 19 of 105 exhibited LOH only at the *RPH3AL* locus. Of these, 12 were non-Hispanic Caucasians (Whites), 15 had large tumors, and 12 were older (>50 years). Patients exhibiting LOH at both loci had shorter survival than those without LOH at these loci (log-rank, P = 0.014). LOH at the *TP53* locus alone was not associated with survival. Analyses of *RPH3AL* identified missense point mutations in 19 of 125 cases, a SNP (C>A) in the 5’untranslated region at -25 (5’UTR-25) in 26 of 104, and a SNP (G>T) in the intronic region at 43 bp downstream to exon-6 (intron-6-43) in 79 of 118. Genotype C/A or A/A of the SNP at 5’UTR-25 and genotype T/T of a SNP at intron-6-43 were predominantly in Whites. Low levels of RNA and protein expression of *RPH3AL* were present in cancers relative to normal tissues. Thus, genetic alterations in *RPH3AL* are associated with aggressive behavior of breast cancers and with short survival of patients.

## Introduction

The human chromosomal region *17p* shows frequent allelic loss/mutations in various cancers, including breast cancer [1–4]. *17p* contains tumor suppressor genes such as *TP53*. Frequently, allelic losses of *17p* coincide with mutations of *TP53* located at *17p13*.*1*. In the absence of genetic alterations in *TP53*, however, breast cancers and other solid tumors exhibit genetic alterations in the telomeric region, including the *17p13*.*3 locus* [5–11]. Such genetic alterations indicate the existence of tumor suppressor genes in addition to *TP53*.

In human medulloblastomas, hemizygous deletion of *17p13*.*3* is associated with poor survival of patients [12]. Smith *et al*. identified the *RPH3AL* gene at the *17p13*.*3* locus (Gene Bank# AF129812) and suggested that it is a human ortholog of the rat *RPH3AL* gene (originally termed *Noc2*) [13]. Human *RPH3AL* exhibits considerable sequence homology with rat *Noc2* (77% identity at the amino acid level) [13]. Although the function of *RPH3AL* in humans is not known, the protein is essential for normal regulation of exocytosis in endocrine and exocrine cells through its interactions with the cytoskeleton [14–17]. RPH3AL was also shown to promote agonist-induced intracellular increase in Ca^2+^ during the exocytosis of zymogen granules in pancreatic acinar cells [16]. Smith *et al*. also cloned, sequenced, and performed mutational analysis of *RPH3AL* gene in medulloblastoma, follicular thyroid carcinoma, and ovarian carcinoma [13]. As these studies failed to identify any missense mutations in *RPH3AL*, they concluded that *RPH3AL* was not involved in the oncogenesis of these neoplasms [13]. However, an earlier investigation of colorectal cancers (CRCs) identified six missense point mutations in the *RPH3AL* gene [18], and studies of CRCs in our laboratory found a single nucleotide polymorphism (SNP) at 5’UTR-25 of *RPH3AL* and demonstrated a strong association between this SNP and short survival of patients [19]. Circulating anti-RPH3AL antibodies have been suggested as a diagnostic biomarker of CRC [20].

Homozygous deletion of *RPH3AL* has been implicated in tumorigenesis of childhood adrenocortical tumors [21]. In Europe, a small study of breast cancers (n = 47) found loss of heterozygosity (LOH) at the *17p13*.*3* locus in 28% of cases; none of these cases exhibited alterations at *TP53* locus [22]. These findings prompted us to evaluate the allelic loss of *RPH3AL*, together with LOH of *TP53*, for clinical utility in breast cancers. Since LOH and SNPs at regulatory regions of genes influence their expression, as observed for thymidylate synthase (*TS*) [23, 24] and *CYP17* [25], we analyzed LOH of *RPH3AL* and evaluated the coding and non-coding regions of *RPH3AL* for mutations and SNPs in breast cancers. The current investigation found previously unidentified missense point mutations and a high incidence of LOH of the *RPH3AL* gene. For breast cancers, the clinical value LOH of the *RPH3AL* gene was established.

## Materials and Methods

### Patients

The Institutional Review Board (IRB) of the University of Alabama at Birmingham (UAB) approved the collection of patient clinical information and utilization of the tissues for these studies. These studies were carried out in compliance with the Helsinki Declaration. All patients had undergone surgery for first primary breast cancer at the UAB hospital and these studies were performed on the remnant tissues of diagnosis. Due to the retrospective nature of the current studies, consent from patients had not been obtained and, as per the guidelines of the IRB of UAB, the patient records/information was anonymized and de-identified prior to analyses. The UAB IRB committee has specifically approved this study.

### Tissue samples

We collected randomly selected, formalin-fixed paraffin embedded (FFPE) tissues from 127 breast cancer patients who had undergone surgical resection for first primary breast cancer from February 1986 through March 2006 at UAB. These histologically confirmed breast cancers and corresponding normal (benign breast epithelial) tissues were used to determine the molecular status of the *RPH3AL* gene. TNM staging of the American Joint Commission on breast cancer was used for tumor staging [26].

### Patient demographics, clinical and follow-up information

Patient demographics, along with clinical and follow-up information, were retrieved retrospectively from medical charts as well as from the UAB Tumor Registry. Patients were followed either by the patient’s physician or by the Tumor Registry until their death or the date of the last documented contact within the study timeframe. The Tumor Registry ascertained outcome (mortality) information directly from patients (or living relatives) and from the physicians of the patients through telephone and mail contacts. This information was verified by the state death registry. Demographic data, including patient age at diagnosis, race/ethnicity, date of surgery, date of the last follow-up (if alive), and date of death were collected. Information on race/ethnicity was obtained from patient charts and this assignment was self-described or self-identified. Follow-up of this retrospective cohort ended in July 2014.

### DNA extraction from paraffin blocks and frozen tissues

Genomic DNA was extracted from archived breast cancers and matching benign epithelial tissues following a previously published method [19, 27]. In brief, for FFPE samples, a 10-μM thick tissue section was deparaffinized in 1 mL of octane (Fisher Scientific, Suwanee, GA). The tissue pellet was re-suspended in 180 μL of digestion buffer [(50 mM Tris, pH 8; 1 mM EDTA; 1% Tween 20) plus 20 μL of proteinase K (20 mg/ml) (Fisher Scientific)], and the mixture was incubated for 24 hr at 56°C. Samples were heated to 95°C, then 200 μL of phenol/chloroform/isoamylalcohol (Fisher Scientific) (25:24:1, pH 6.7) was added. The aqueous layer was transferred into Microcon YM-100 filter tubes (Fisher Scientific, USA). The DNA was eluted from the filter tubes by adding 125 μL of TE buffer (10 mM Tris hydrochloric acid; 0.1 mM EDTA, pH 8). DNA quality and concentration were determined by spectrophotometry. Genomic DNA from snap-frozen tissues was extracted by use of DNeasy Tissue Kits (Qiagen). The quality of DNA, defined by the ratio of E260nm/280nm = 1.8–2.0, was ascertained for all samples using NanoDrop (Fisher Scientific).

### LOH analysis of *RPH3AL* and *TP53*

Two polymorphic microsatellite markers at the *17p13*.*3* locus in *RPH3AL* (*D17S1866*, *D17S643*) were used to determine the LOH status of the *RPH3AL* gene. Also, the LOH status of *TP53* was evaluated by use of two polymorphic microsatellite markers at the *17p13*.*1* locus (TP53.PCR15, TP53.PCR18) in the same set of DNA samples. The primer sequences of these four markers given in the database of NCBI were [(D17S1866-forward-TGGATTCTGTAGTCCCAGG and reverse-GGTTCAAAGACAACTCCCC; D17S643-forward-CTTCCTTGTCTCTAAACAGTCCTTT and reverse-GTAGTCCCAGGGAGCTGGAAGT; TP53.PCR15-forward-AGGGATACTATTCAGCCCGAGGTG and reverse-ACTGCCACTCCTTGCCCCATTC; and TP53.PCR18-forward-TTCCGCAGTTTCTTCCCATG; and reverse-TGTGTGTAAATGCCACCTCG). In each primer set, the forward primer was labeled with fluorescent dye for allele detection (Applied Biosystems, Inc., Foster City, CA). The PCR reaction mixture (25 μL) consisted of 1×PCR buffer, 10 mM dNTPs, 15 mM MgCl_2_, 10 pmoles of each primer, 0.3 μL (2.5 units) of Platinum Taq Polymerase (Life Technologies, Grand Island, NY), and 100 ng of genomic DNA. Amplification was achieved by 2 min of initial denaturation at 95°C, followed by 35 cycles each with a 30-sec denaturation at 95°C, 30 sec annealing at 60°C, and 1 min extension at 72°C. Final extension was for 20 min at 72°C. Each labeled PCR product (2 μl) was added to the mixture of 12 μl of deionized formamide and 1 μl of Gene Scan 500 ROX (Life Technologies) and then denatured at 88°C for 5 min, followed by chilling on ice for 2 min and centrifuging for 15 sec. Microcapillary electrophoresis of PCR products was accomplished with an ABI 3100 genetic analyzer (Life Technologies). The data were collected automatically and analyzed by Genotyper 2.1 software (Life Technologies). LOH was defined for each tumor as α = (TL1 x NL2)/(TL2 x NL1) where L is the intensity of allele 1 or 2 in normal (N) or tumor (T) DNA. An α-score ≤ 0.5 or ≥1.5 was defined as LOH positivity. Homozygous cases were considered non-informative for LOH.

### *RPH3AL* sequence analysis

#### Frozen Cohort

To assess the mutational status of *RPH3AL* by use of the reverse transcription-polymerase chain reaction (RT-PCR) and DNA sequencing methods, 28 frozen specimens of breast cancer and their matching benign breast tissues were analyzed. The direct DNA sequencing method using *RPH3AL* specific primers (forward 5’-3’: GTGCACTTTGG AGACAGCAA and reverse 5’-3’: GTGGGAGGGGA GGGT AATAA) resulted in a cDNA transcript, which covered exons 1 through 9. Since earlier studies suggested that the putative start codon was in the middle of the exon 2 [13], our cDNA transcript also covered the untranslated regions of exons 1 and 2.

#### FFPE Cohort

Since a preliminary study of the frozen cohort of breast cancer tissues as well as our results with CRCs demonstrated missense point mutations only in exon-6 (5 of 28, ~18%) (S1 Table), sequence analyses for the FFPE cohort were focused on evaluating exon-6 and the down-stream part of intron-6 (referred to as exon-6) of *RPH3AL*. The following primers were used to amplify this region: forward primer- 5’ cccactcaggtctggaagag 3’ and reverse primer- 5’ gagagaggcagaggggactt 3’. The standard reaction mixture (25 μl) contained 100 ng of genomic DNA, 0.25 μmol/L of each primer, 0.2 mmol/L of each dNTP, 1X PCR buffer, 2 mmol/L MgCl_2_, and 0.5 units of Platinum Taq DNA polymerase (Invitrogen). PCR was performed on a Robocycler (Agilent Technologies Inc., Santa Clara, CA) using a program with initial denaturation at 94°C for 10 min followed by 35 cycles, each with 30 sec denaturation at 94°C, 30 sec annealing at 55°C, and 1 min extension at 72°C. The final extension step was 7 min at 72°C. The purified PCR products were directly sequenced on a ABI 3100 sequencer (Life Technologies). Forward and reverse strands of exon-6 from both tumor and normal/benign tissues were sequenced.

### Genotyping of the SNP at 5′UTR-25 of *RPH3AL*

Genomic DNA samples were analyzed for the genotype at the 5′UTR-25 SNP utilizing the PCR-confronting, two-pair primer (PCR-CTPP) method that we have used for genotyping of this SNP [19]. In brief, the amplification of allele-specific bands of different lengths was accomplished using four primers for genotyping by electrophoresis. The four primers consisted of F1 and R1 for the amplification of one allele, and F2 and R2 for the amplification of the other allele. F1 and R2 produce a common PCR product that is independent of the difference in alleles. F2 and R1 confront each other at the 3′ end with the base specific to the allele. The primers designed to detect the 5′UTR-25 C to A variant were: F1 (5′-GAGGGCACAGAGAACCTGTC-3′), R1 (5′-GGAGCACCCGGCTGGGGGTT-3′), F2 (5′-CATCTCAGATGTGACTCCCC-3′), and R2 (5′-GGCCCCAGAGGTACTCACTT-3′). PCR products were fractionated by electrophoresis in a 2% agarose gel and stained with ethidium bromide. To confirm the genotype of 5′UTR-25, the products of PCR-CTPP were analyzed by direct DNA sequencing.

### Quantitative real time polymerase chain reaction (qRT-PCR) analysis

Total RNA was extracted from 127 breast cancers and their matching normal tissues by use of RNeasy Kits (QIAGEN), and 1 μg of total RNA was reverse-transcribed to cDNA. Quality of RNA was assessed by the ratio of absorbance at 260 nm and 280 nm using NanoDrop (Fisher scientific) and a 260/280 value of ~2 was ensured for the samples used in our experiments. Quantitative RT-PCR was performed in a volume of 25 μL, consisting of 0.5 μL of each primer (5 pmoles), 12.5 μL of 2x Supermix containing the reaction buffer, Fast-start Tag DNA double strand-specific SYBR green I dye, 6.5 μL of nuclease-free water, and 5 μL of cDNA template. Quantitative RT-PCR was accomplished by means of a program with initial denaturation at 95°C, followed by 45 cycles each with 15 sec denaturation at 95°C, and 30 sec annealing and extension at 60°C. The PCR reactions were performed on an i-Cycler Real-time PCR system (Bio-Rad Laboratories, Hercules, CA) with β-actin and *RPH3AL* primer sets. PCR products were subjected to melting curve analysis to exclude non-specific amplification. The PCR reactions were performed in sets of four. Means of *RPH3AL* mRNA and β-actin mRNA copy numbers were calculated for each case separately, and ratios of these means were calculated.

### Immunohistochemical analysis

Breast cancer FFPE tissue sections of 5-μm thickness were cut and mounted on Superfrost/Plus slides (Fisher Scientific, Pittsburgh, PA). Immunostaining was conducted as described earlier [28, 29]. Briefly, the sections were incubated at 60°C for 1 hr, followed by overnight incubation at 37°C. The tissue sections were deparaffinized in xylene and rehydrated through graded alcohols. The sections were transferred to a buffer bath (0.05 M Tris base, 0.15 M NaCl, and 0.01% Triton X-100, pH 7.6). Antigen retrieval was performed on the sections by use of a pressure cooker for 10 min with EDTA buffer, 0.01 M at pH 9 (2.1 g EDTA, 1000 ml H_2_O, adjusting pH with NaOH). Tissue sections were treated with 3% H_2_O_2_ for 5 min. Tissues were blocked with 3% goat serum at room temperature for 1 h followed by incubation with anti-human RPH3AL antibody (rabbit polyclonal, 5 μg/ml, developed by our laboratory). Sections were then incubated in horseradish peroxidase-labeled secondary antibody and visualized by diaminobenzidine detection. The sections were further counterstained with hematoxylin, dehydrated through graded alcohols, and soaked in xylene before cover-slipping.

### Statistical analyses

The χ^2^-test and Fisher’s exact test were used to compare baseline characteristics as described [30]. Deaths due to breast cancer were the outcomes (events) of interest. The prognostic significance of LOH at the *17p13*.*3* locus of *RPH3AL* and the *17p13*.*1* locus of *TP53* was analyzed by the Kaplan-Meier procedure [31]. For survival analyses, the risk of breast cancer-specific death was measured by calculating the number of months from the date of surgery to death or to the date of last contact. Patients who died of a cause other than breast cancer or who were alive at the end of the follow-up period were “right censored.” Survival analysis was accomplished, and log-rank tests were used to compare Kaplan-Meier curves based on the LOH status. All analyses were performed with SAS statistical software, version 9.3 [32], [33]. P-values were calculated, and significance was analyzed at an α level of 0.05.

Cox proportional hazards models were generated to obtain unadjusted bivariate and adjusted multivariate hazard ratios (HR) with 95% confidence intervals (CIs) for the association of covariates with cancer specific mortality. All models were tested for and met assumptions of proportionality. Variable selection for models was based on clinical importance and guided by significance in univariate analysis as well as sample size and missing values. Cox survival models were built by including *RPH3AL* LOH status and the known confounding covariates (age, race/ethnicity, tumor stage, size, and histologic grade, as well as ER/PR status). However, we have not controlled for other confounding variables such as socioeconomic status, nutrition, and environmental exposures in our analyses.

## Results

### Clinicopathological features of the study cohort

At the time of surgery, the mean age of the patients in the study cohort was 55 years (range, 27–82 years). There was a preponderance of Whites (77 of 126, 61%), and Stage II cancer cases (57 of 121, 47%) (Table 1). At the last follow-up, 48% (61 of 127) of the patients were alive. Cancer-related death was reported for 24% (30 of 127), relative to 13% (17 of 127) non-cancer related deaths.

**Table 1:** Characteristics of the study cohort.

Variable^1^	Number of cases (%)
**Age** (years) (*N* = 127)	** **
≤ 50	46 (36)
>50	81 (64)
**Ethnicity** (*N* = 126)	** **
White	77 (61)
Black	49 (39)
**Tumor size** (*N* = 127)	** **
≤ 2 cm	50 (39)
> 2 cm	77 (61)
**ER/PR status** (*N* = 119)	
Positive ^2^	77 (65)
Negative	42 (35)
**Nodal status** (*N* = 117)	
Positive	55 (47)
Negative	62 (53)
**Tumor grade** (*N* = 96)	** **
I	19 (20)
II	23 (24)
III	54 (56)
**Tumor stage** (*N* = 121)	
in-situ	3 (2)
I	31 (26)
II	57 (47)
III & IV	30 (25)

ER, estrogen receptor; PR, progesterone receptor

^1^Not all patients had information available for all the clinicopathological features evaluated

^2^Either or both receptors positive.

### Mutational status of *RPH3AL* in breast cancer

Our initial proof-of-concept study on a small cohort of frozen breast cancer cases found mutations only in exon-6. As shown in S1 Table, 21% (6 of 28) of breast cancer cases exhibited mutations in the *RPH3AL* gene. Five of these mutations were missense point mutations at codon 175, resulting in amino acid substitutions. One of six mutated cases exhibited SNPs at codons 49 and 62. Two breast cancer cases that exhibited missense point mutations at codon 175 also exhibited a silent mutation at codon 62.

Since our preliminary sequence analysis of *RPH3AL* showed mutations confined to exon-6, further sequencing of *RPH3AL* in 125 FFPE breast cancer specimens focused only on exon-6. The *RPH3AL* mutational status of the FFPE cohort is summarized in Table 2. Missense point mutations in the coding region of exon-6 of *RPH3AL* were present in 19 of 125 (15%) of breast cancers. Most breast cancers had single mutations. However, 3 cases had double mutations, and one case had three mutations (Table 2). Ten breast cancers exhibited mutations at codon 175, and 3 breast cancers had mutations at codon 165. Three breast cancers had single mutations at codons 174, 181, and 196; and 2 breast cancers had mutations at codon 200. Mutations at codons 174, 175, 181, 196, or 200 were missense point mutations resulting in amino acid substitutions, whereas mutations at codon 168 and 189 were silent mutations. Twelve of 19 (67%) cases with *RPH3AL* mutations were also positive for *RPH3AL* LOH. There was no significant association between the missense point mutations and clinicopathological features. Although the sample size was small, patients with *RPH3AL* LOH and with missense mutations had poor survival relative to the patient group with mutant *RPH3AL* but negative for LOH (S1 Fig).

**Table 2:** Genetic abnormalities of the *RPH3AL* gene and demographic and pathological features of breast cancers.

	*RPH3AL* mutations								
Case number	Codon number	Nucleotide change	Amino acid change	Stage	Grade	Nodal status	ER/PR	*RPH3AL* LOH	*TP53* LOH
BC-5	178	CGA>TGA	Arg>Stop	II	III	N	NA	P	P
	189	CCC>CCT	Pro>Pro						
BC-21	181	CCC>TCC	Pro>Ser	II	III	NA	N	P	P
BC-24	174	GAC>AAC	Asp>Asn	II	III	N	N	P	P
BC-30	196	CGC>TGC	Arg>Cys	III	III	P	N	P	P
BC-31	200	TGG>CGG	Trp>Arg	I	I	N	P	N	P
BC-32	175	CCC>TCC	Pro>Ser	III	II	P	N	P	N
BC-40	200	TGG>TGA	Trp>Stop	III	II	P	NA	P	P
BC-41	165	CCC>CCT	Pro>Pro	III	III	P	NA	P	N
BC-50	175	CCC>TCC	Pro>Ser	II	III	N	N	P	P
BC-52	165	CCC>TCC	Pro>Ser	I	III	N	P	P	P
BC-53	175	CCC>TCC	Pro>Ser	II	II	N	N	N	N
BC-55	159	CTC>CTT	Leu>Leu	III	II	P	P	P	P
	175	CCC>TCC	Pro>Ser						
BC-58	165	CCC>CCT	Pro>Pro	II	III	N	N	N	P
	175	CCC>TCC	Pro>Ser						
	181	CCC>CTC	Pro>Leu						
BC-61	168	ACC>ACT	Thr>Thr	I	I	N	N	P	N
	189	CCC>CCT	Pro>Pro						
BC-91	175	CCC>CTC	Pro>Leu	II	III	P	P	N	N
BC-92	175	CCC>CTC	Pro>Leu	II	III	P	P	N	P
BC-94	175	CCC>CTC	Pro>Leu	II	III	P	N	N	P
BC-104	175	CCC>TCC	Pro>Ser	II	III	P	NA	P	P
BC-140	175	CCC>TCC	Pro>Ser	III	III	P	N	NA	NA

ER, estrogen receptor; PR, progesterone receptor; LOH, loss of heterozygosity; P, either or both receptors positive; N, both receptors negative; NA, not available.

We previously identified a SNP in *RPH3AL* 5’UTR at -25 and demonstrated its clinical significance in CRC [19]. Analysis of 104 FFPE breast cancer tissues for 5’UTR-25 SNP showed genotypes C/C in 78 (75%), C/A in 24 (23%), and A/A in 2 (2%) cases. The 5’UTR-25 SNP was also significantly associated with patient ethnicity (*χ* ^2^ P = 0.013) and histologic grade of the tumor (*χ* ^2^ P = 0.032) (Table 3). Sequence analysis showed another SNP at 43 bp downstream to exon-6 in the intron-6 region (intron-6-43). The intron-6-43 SNP resulted in a change in the nucleotide from guanine to thymine (transversion). The genotype of the intron-6-43 SNP and its correlation with clinicopathological features is illustrated in Table 3. Analysis of 118 breast cancers for the intron-6-43 SNP demonstrated that the genotypes G/G, G/T, and T/T were found in 39 (33%), 64 (54%), and 15 (13%) breast cancers, respectively. The SNP was strongly associated with patient ethnicity (*χ* ^2^ P = 0.002), with Whites predominantly exhibiting the T/T genotype (14 of 15, 93%) (Table 3).

**Table 3:** Relationship between clinicopathological features and SNPs at 5’UTR-25 and intron-6-43 of *RPH3AL* in breast cancers.

	5' UTR-25 Genotype				Intron-6-43 Genotype			
	C/C	C/A	A/A		G/G	G/T	T/T	
	*N = 78*	*N = 24*	*N = 2*		*N* = 39	*N* = 64	*N* = 15	
Characteristic^1^	*N* (%)	*N* (%)	*N* (%)	P-value	*N* (%)	*N* (%)	*N* (%)	P-value
**Age Group (years)**								
≤ 50	26 (70)	10 (27)	1 (3)		16 (36)	22 (50)	6 (14)	
> 50	52 (78)	14 (21)	1 (1)	0.602	23 (31)	42 (57)	9 (12)	0.774
**Ethnicity**								
White	42 (66)	20 (31)	2 (3)		17 (23)	42 (58)	14 (19)	
Black	35 (90)	4 (10)	0 (0)	**0.013**	22 (50)	21 (48)	1 (2)	**0.002**
**Tumor size**								
≤ 2 cm	33 (71)	12 (26)	1 (2)		13 (28)	28 (60)	6 (12)	
> 2 and ≤ 5 cm	38 (79)	9 (19)	1 (2)		19 (35)	27 (49)	9 (16)	
> 5 cm	7 (70)	3 (30)	0 (0)	0.83	7 (44)	9 (56)	0 (0)	0.387
**Histologic grade**								
I	9 (60)	5 (33)	1 (7)		3 (18)	12 (75)	1 (6)	
II	15 (68)	7 (32)	0 (0)		10 (45)	9 (41)	3 (14)	
III	37 (88)	5 (12)	0 (0)	**0.032**	17 (34)	25 (50)	8 (16)	0.292
**ER/PR status**								
Positive	50 (74)	17 (25)	1(1)		26 (36)	39 (54)	7 (10)	
Negative	24 (77)	6 (19)	1 (3)	0.077	9 (23)	24 (60)	7 (17)	0.232
**Nodal status**								
Positive	35 (76)	10 (22)	1(2)		21 (42)	24 (48)	5 (10)	
Negative	36 (74)	12 (24)	1 (2)	0.904	15 (26)	34 (59)	9 (16)	0.193
**Tumor Stage**								
I	19 (64)	10 (33)	1 (3)		7 (23)	21 (68)	3 (10)	
II	34 (79)	8 (19)	1 (2)		16 (29)	29 (53)	10 (18)	
III & IV	21 (84)	4 (16)	0 (0)	0.379	13 (50)	11 (42)	2 (8)	0.156^2^

^1^Not all patients had information available for all the clinicopathological features

^2^Fisher exact

### Expression of *RPH3AL* in breast cancer tissues

**Fig 1:**
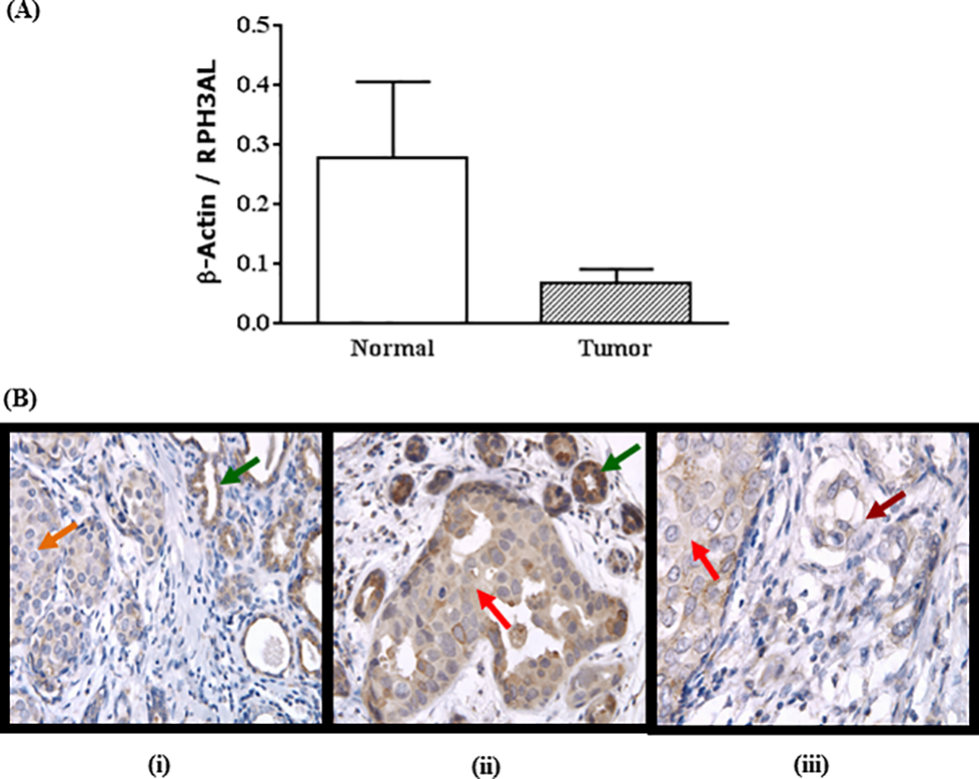
Down-regulation of *RPH3AL* mRNA and protein in breast cancers. Analyses of mRNA and protein expression of *RPH3AL* **(A)** mRNA levels of *RPH3AL* down-regulated in breast cancers relative to their matching normal tissues. **(B)** Immunohistochemical staining of RPH3AL in breast cancer tissues. RPH3AL expression was seen predominantly in the cytoplasm of **(i)** lobular intraepithelial neoplasia (LIN, orange arrow) and adjacent normal cells (green arrow) (X100) **(ii)** ductal carcinoma in situ (DCIS, red arrow) and adjacent normal cells (green arrow) (X100) **(iii)** DCIS (red arrow) and invasive carcinoma (dark red) (X200). LIN and DCIS exhibit lower expression of RPH3AL relative to adjacent normal cells. Mild nuclear staining was also seen in a few cells of normal, LIN, DCIS, and invasive carcinoma.

Gene expression analysis demonstrated that the mRNA levels of *RPH3AL* were down-regulated in breast cancers relative to their matching normal tissues (Fig 1A). The immunophenotypic expression pattern of RPH3AL in breast cancer tissues, determined by IHC, was consistent with mRNA expression levels of *RPH3AL*. The malignant cells of lobular intraepithelial neoplasia (LIN), ductal carcinoma in situ (DCIS), and invasive carcinoma exhibited lower expression of RPH3AL protein relative to normal cells in benign ducts and stroma (Fig 1B). RPH3AL immunostaining was seen predominantly in the cytoplasm of both tumor and adjacent normal breast tissues, although the extent and intensities of staining varied between malignant and non-malignant cells. There was mild nuclear staining in a few tumor cells and normal cells (Fig 1B).

### LOH status of *RPH3AL* and its correlations with clinicopathological features and LOH status of *TP53*

Associations between clinicopathological characteristics and LOH status are shown in Table 4. In our analyses, 121 of 127 were informative for LOH; the remaining 6 cases were homozygous at the loci. There was a higher frequency of breast cancers with LOH (LOH positive) (80 of 121, 66%) than without LOH (LOH negative) (41 of 121, 34%). The incidence of LOH positivity was higher in tumors with large size (56 of 73, 77%, χ^2^ P = 0.002), nodal metastasis (38 of 51, 75%, χ^2^ P = 0.049), and advanced stage (24 of 28, 86%, χ^2^ P = 0.013) (Table 4). About 18% (19 of 105 informative) cases were positive for LOH of *RPH3AL* but negative for LOH of *TP53*. Most of these patients were Whites (12 of 19) with large size tumors (15 of 19) (P = 0.042) and who were older (>50 years) (12 of 19) at the time of diagnosis (S2 Table).

**Table 4:** Relationship between clinicopathological features and LOH status at *17p13*.*3* of the *RPH3AL* gene in breast cancers.

Characteristic^1^	LOH+	LOH-	P-value
	*N* = 80	*N* = 41	
	*N* (%)	*N* (%)	
**Age Group (years)**			** **
≤ 50	24 (59)	17 (41)	** **
> 50	56 (70)	24 (30)	0.207
**Ethnicity**			
White	46 (64)	26 (36)	
Black	33 (69)	15 (31)	0.582
**Tumor size**			
≤ 2 cm	24 (50)	24 (50)	
> 2 cm	17 (23)	56 (77)	**0.002**
**Histologic grade**			
I	11 (61)	7 (39)	
II	14 (64)	8 (36)	
III	35 (66)	18 (34)	0.927
**ER/PR status**			
Positive	43 (59)	30 (41)	
Negative	30 (75)	10 (25)	0.087
**Nodal status**			
Positive	38 (75)	13 (25)	
Negative	34 (57)	26 (43)	**0.049**
**Tumor Stage**			
I	17 (50)	17 (50)	
II	35 (66)	18 (34)	
III&IV	24 (86)	4 (14)	**0.013** ^**2**^
***TP53* LOH**			** **
Positive	52 (73)	19 (27)	** **
Negative	19 (56)	15 (44)	0.075

^1^Not all patients had information available for all the clinicopathological features

^2^Fisher's exact

### Prognostic value of LOH of *RPH3AL* alone and in combination with LOH of *TP53*

**Fig 2:**
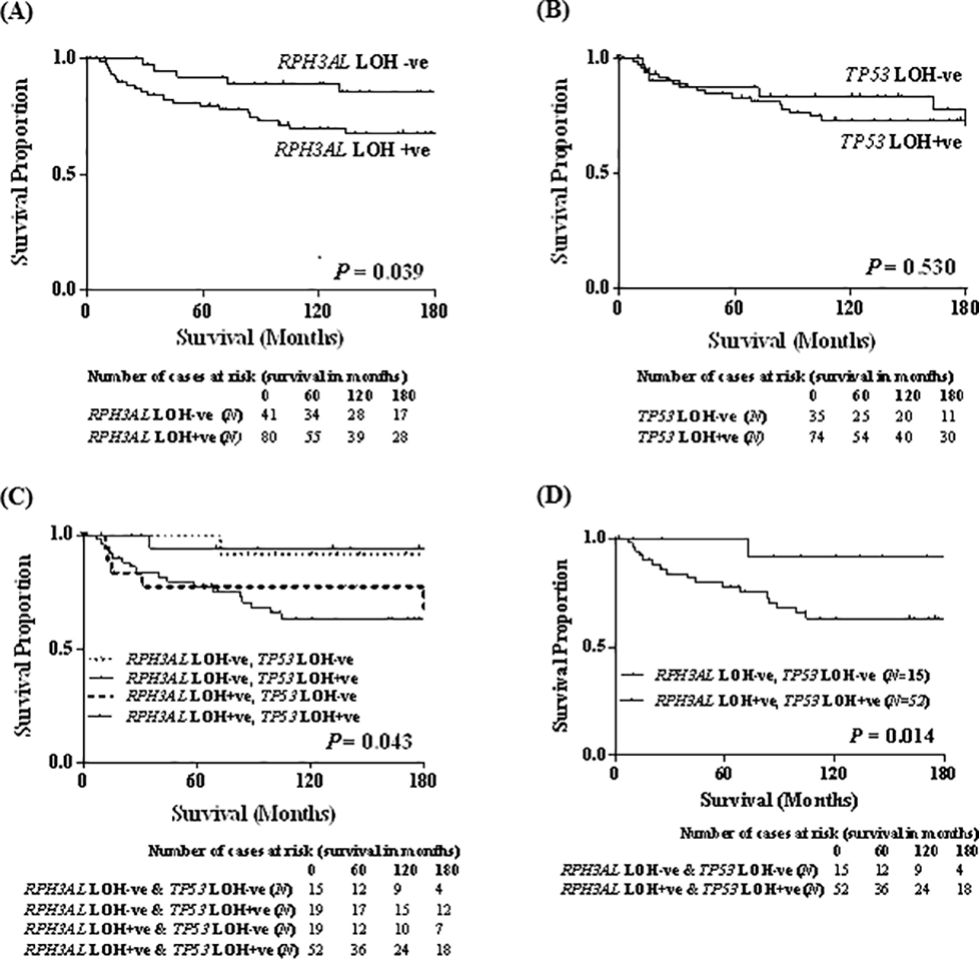
Prognostic significance of *RPH3AL* LOH. Kaplan-Meier survival analyses of **(A)** LOH at *17p13*.*3* locus of *RPH3AL*, **(B)** LOH at *17p13*.*1* locus of the *TP53*, and **(C)** combinations of LOH at *RPH3AL* and *TP53*, and **(D)** survival proportions of patients with LOH at both *RPH3AL* and *TP53* loci *versus* the patients without LOH at both loci. Log-rank P-values and the number of patients at risk (*N*) in each group at different time periods. Positivity of *RPH3AL* LOH, but not *TP53* LOH, was associated with patient poor survival. Patients with LOH positivity at both *RPH3AL* and *TP53* loci had poor survival relative to patients with LOH at *TP53* and without LOH at *RPH3AL* (P = 0.008) as well as those that were LOH negative at both loci (P = 0.014). Also, the subgroup with LOH positive at *RPH3AL* and LOH negative at *TP53* locus had poor survival, similar to the patients that were positive at both loci (P = 0.101), suggesting that *RPH3AL* LOH is associated with patient prognosis.

Kaplan-Meier analysis of the breast cancers demonstrated that patients with LOH-positive tumors had poor survival relative to patients with LOH-negative tumors (log rank, P = 0.039) (Fig 2A). Since *TP53*, located in proximity to *RPH3AL* within the *17p* chromosomal region, is a driver gene and exhibits LOH in breast cancers, the *TP53* LOH status of breast cancer cohorts was analyzed. Of the 127 cases analyzed for LOH status at the *17p13*.*1* locus of *TP53*, 105 cases were informative for LOH, and 22 cases were homozygous at the loci. In the FFPE cohort, there was no significant difference in the survival probability between *TP53* LOH-positive and-negative patient groups (log rank, P = 0.530) (Fig 2B).

Univariate survival analysis, considering the combination of LOH status of *RPH3AL* and *TP53* loci, revealed a significant difference in the survival proportions of the four categories (log rank, P = 0.043) (Fig 2C). The patients with LOH at both loci had poor survival relative to patients without LOH at these loci (log rank, P = 0.014) (Fig 2D). Also, patients without LOH at *RPH3AL* and with LOH at *TP53* locus had better survival relative to those with LOH at both loci (log rank, P = 0.008).

Although univariate survival analysis showed a significant association of *RPH3AL* LOH with poor survival of patients, Cox regression analyses (unadjusted and adjusted) suggested that the LOH status of *RPH3AL* is not a significant predictor of the hazard of cancer-related death (HR = 1.53, 95% CI: 0.66–3.65; P = 0.331 for adjusted analysis) (Table 5). Positive nodal status, tumor size, and late stage of the disease were significant variables in the unadjusted analysis; in the adjusted model, only positive nodal status remained as a significant predictor of hazard of cancer-related death (HR = 4.76, 95% CI: 1.96–11.55; P = 0.001) (Table 5).

**Table 5:** Cox regression analyses.

	Unadjusted		Adjusted	
Variable	HR (95% CI)	P-value	HR (95% CI)	P-value
**Age Group (years)**	** **	** **		
> 50	Ref		Ref	
≤ 50	1.76 (0.88–3.48)	0.106	1.67 (0.80–3.47)	0.170
**Ethnicity**				
White	Ref		Ref	
Black	1.32 (0.65–2.70)	0.449	1.48 (0.67–3.30)	0.336
**Tumor size**				
≤ 2 cm	Ref		Ref	
> 2 cm	2.21 (1.02–4.76)	**0.043**	1.20 (0.50–2.85)	0.687
**Histologic grade**			-	-
I	Ref			
II	1.22 (0.33–4.56)			
III	1.44 (0.47–4.44)	0.805		
**ER/PR status**			-	-
Positive	Ref			
Negative	0.80 (0.39–1.68)	0.563		
**Nodal status**				
Negative	Ref		Ref	
Positive	4.28 (1.90–9.67)	**0.001**	4.76 (1.96–11.55)	**0.001**
**Tumor Stage**			-	-
I	Ref			
II	1.80 (0.57–5.64)			
III & IV	9.63 (3.12–29.10)	**<0.0001**		
**RPH3AL LOH**				
LOH -	Ref		Ref	
LOH +	2.17 (0.94–5.03)	0.070	1.53 (0.66–3.65)	0.331

HR, hazard ratio; CI, confidence interval; Ref, reference group.

## Discussion

The current study identified a high proportion (66%) of breast cancers with LOH at a *RPH3AL* locus and a low incidence of missense point mutations (15%), found only in exon-6. Also, a SNP at intron-6-43 was identified. The genotype T/T was predominantly found in White patients. This study also confirmed the identity of a previously known SNP at 5′UTR-25 and suggested that homozygosity for the A allele was found only in White patients. Consistent with the nature of a tumor suppressor, mRNA and protein expressions of RPH3AL were low in breast cancer tissues relative to their corresponding control/benign tissues. The higher incidence of LOH correlated with large tumor size, node positivity, and advanced tumor stage. In univariate analyses, LOH of *RPH3AL* was associated with poor survival of patients, and patients who exhibited LOH at both *RPH3AL* and *TP53* loci had shorter survival than those without LOH at these loci. A small group of patients (n = 19) who exhibited LOH of *RPH3AL* but not *TP53* were predominantly Whites, with large size tumors, and were older at the time of breast cancer diagnosis. Thus, the LOH status of *RPH3AL* augments the use of nodal involvement in predicting the risk of breast cancer-specific death.

In human malignancies, LOH is a genetic abnormality having a profound effect on changes in allele copy numbers and the level of expression of various tumor suppressor genes [34]. LOH is also associated with tumor progression and poor survival [35]. Several tumor suppressor genes that are located on chromosome 17 may contribute to tumorigenesis of breast cancer. Two independent regions of allelic loss on chromosome *17p* in breast tumors, one spanning *TP53* and the other involving a telomeric region, imply the existence of another tumor suppressor gene distal to *TP53* [1]. For breast cancers, there are associations between allele loss at *17p* and a high proliferation index [36] and poor survival of patients [37]. Therefore, the current study focused on evaluating abnormalities at the *17p13*.*3* locus, where the *RPH3AL* gene is located. Since *TP53* is located in proximity to *RPH3AL* on *17p* and since LOH of *TP53* is involved in development of various cancers, including breast cancer, LOH of *TP53* at the *17p13*.*1* locus [38–40] was also assessed.

The present results show LOH at the *17p13*.*3* locus of *RPH3AL* in 66% of cases and that, in univariate analyses; it is associated with larger tumor size, nodal metastasis, advanced tumor stage, and poor patient survival. Although a similar incidence of LOH at *TP53* was observed (68%), *TP53* LOH alone did not show a significant association with any of these clinical features or with patient survival. A small study of breast cancers (n = 47) in Europe found LOH at the *17p13*.*3* locus in 28% of cases, but none of these cases had alterations at the *TP53* locus, and LOH of *17p13*.*3* correlated with aggressive tumor features [22]. In our study, patients with LOH at the *RPH3AL* locus and without LOH at the *TPP53* locus had poor survival. Moreover, patients without LOH at *RPH3AL* and with LOH at *TP53* loci had significantly better survival relative to patients with LOH at both loci. These findings show that allelic loss of *RPH3AL* is a strong prognostic indicator, independent of *TP53* allelic loss. Also, our findings suggest that there is a need to increase the sample size to establish an independent prognostic value of allelic loss of *RPH3AL* in breast cancer.

In various cancers, decreased expression of tumor suppressor genes is a common molecular event influenced by allelic loss or epigenetic mechanisms. Our previous studies showed that *RPH3AL* mRNA expression was decreased in CRCs and suggested its tumor suppressor role in these cancers [19]. Consistent with previous results, the present study found lower expression of *RPH3AL* mRNA in breast cancers relative to matching normal tissues. We and others previously reported immunohistochemical expression of RPH3AL protein in normal and tumor tissues of human endocrine pancreas [41] and bladder cancers [42]. Consistently, in breast cancer tissues, there were lower levels of RPH3AL protein in malignant breast cells (LIN, DCIS, and invasive carcinoma) relative to nonmalignant cells.

Tumor development is characterized by inactivation of tumor suppressor genes, such as *TP53*, by various mechanisms, including mutations. These mutations can cause functional loss of tumor suppressor genes, which in turn promotes tumor progression and leads to poor survival of patients [43]. Mutations in the *RPH3AL* gene are implicated in tumorigenesis of CRCs [18, 19]. The present study identified missense point mutations in 15% of breast cancers. Most of the cases with mutations in *RPH3AL* were also positive for LOH (12 of 18, 67%). Consistent with the Knudson two-hit hypothesis of tumorigenesis [44], these cases have overall poor survival relative to *RPH3AL* LOH-positive cases without mutations (P = 0.053). These results suggest that mutations within the *RPH3AL* gene relate to the pathogenesis of breast cancer and point to a function of *RPH3AL* as a tumor suppressor gene in breast cancer.

Evaluation of SNPs, which are common DNA sequence variations among individuals, may enhance our ability to understand and treat human diseases, including cancer [45]. SNPs, located particularly in gene promoters and gene encoding regions, may influence gene function and/or transcriptional efficiency [45]. For various human genes, SNPs in non-coding regions are implicated in mRNA transcription, stability, and expression, as observed for the *TS* [23] and *CYP17* genes [25]. In other oncogenes, SNPs or somatic mutations in either 3’ or 5’UTRs correlate with alterations in mRNA translation [46], tumor size [47], tumor susceptibility, tumor metastasis, and poor survival [48]. As revealed in our investigation, the overall prevalence of the genotype C/C at 5’UTR-25 in the *RPH3AL* gene was high. Based on these findings, we suspect that alterations in the 5’UTR-25 of *RPH3AL* influence its capacity for transcription and/or translation. Thus, these changes may influence its expression and its function in the regulation of downstream molecular events that are favorable for aggressiveness of neoplasia.

DNA polymorphisms and their incidences, susceptibility for occurrence of genetic alterations, and the risk of tumor progression for patients with cancer vary substantially between racial groups [49, 50]. Although most polymorphisms are functionally neutral, some affect regulation of gene expression or the function of the coded protein. These functional polymorphisms, despite being of low occurrence, could contribute to the differences between individuals or races in susceptibility and severity of disease [49, 50]. In our previous study, a higher prevalence of the mutant genotype at the 5’UTR-25 of *RPH3AL* and its association with a poor prognosis of CRC in White patients was noted, suggesting that there is substantial inter-individual variation in susceptibility to genetic events and to tumor development [19]. In the present study, the mutant A/A genotype exclusively and the mutant C/A genotype with higher frequency was observed in breast cancers of White patients. Similarly, the T/T genotype of the intron-6-43 SNP was observed at higher frequency in White patients. These findings suggest that the SNPs of *RPH3AL* are race-specific molecular markers for White patients with breast cancers. Of note, the missense mutations at codons 165, 174, 181, 196, and 200 and the SNP in intron-6-43 of the *RPH3AL* gene have not been previously reported for human malignancies. While a long follow-up period of the study population is strength of this study, lack of complete information for some of the clinicopathological features (e.g., histologic grade) is a limitation.

## Conclusions

In conclusion, the correlations found between genetic alterations of *RPH3AL* with aggressive features of cancer indicate that genetic abnormalities of the *RPH3AL* gene are involved in breast cancer progression. Although these correlations need to be validated in large prospective studies, our findings suggest that, together with other confounding factors of disease progression, analysis of the molecular status of the *RPH3AL* gene would aid in understanding the aggressiveness of a sub-set of breast cancers and in designing optimal treatment regimens.

## Supporting Information

S1 Fig LOH of *RPH3AL* but not the missense mutations contribute to patient poor survival. (DOCX)

S1 Table Genetic abnormalities of the *RPH3AL* gene and demographic and pathological features of frozen breast cancer tissues. (DOCX)

S2 Table Relationship between clinicopathological features and the combinations of *RPH3AL* and *TP53* LOH status in breast cancers. (DOCX)
