# Supplementary material for: Clinical Implications of Rabphillin-3A-Like Gene Alterations in Breast Cancer
Source: PLoS One. 2015 Jun 12;10(6):e0129216. doi: 10.1371/journal.pone.0129216 (PMC4466565; doi:10.1371/journal.pone.0129216)
Supplement: S1 Fig — (DOCX) [file pone.0129216.s001.docx]

**S1 Fig.** Kaplan-Meier survival analysis of patients exhibiting mutations in *RPH3AL* and LOH at *17p13.3* locus of *RPH3AL versus* the patients with mutations in *RPH3AL* and without LOH at this locus. Patients who exhibited both mutation and LOH in *RPH3AL* had poor survival relative to patients with mutation and without LOH at *RPH3AL* locus (Log-rank, P=0.053).
