# Supplementary material for: Clinical Implications of Rabphillin-3A-Like Gene Alterations in Breast Cancer
Source: PLoS One. 2015 Jun 12;10(6):e0129216. doi: 10.1371/journal.pone.0129216 (PMC4466565; doi:10.1371/journal.pone.0129216)
Supplement: S1 Table — (DOCX) [file pone.0129216.s002.docx]

**S1 Table**: Genetic abnormalities of the *RPH3AL* gene and demographic and pathological features of frozen breast cancer tissues.

|  |  | ***RPH3AL* mutations** | |  |  |  |  |
| --- | --- | --- | --- | --- | --- | --- | --- |
| **Case number** | **Codon number** | **Nucleotide change** | **Amino acid change** | **Stage** | **Tumor Grade** | **Nodal Status** | ***RPH3AL* LOH** |
| FBC1 | 49^1^  62^1^ | CCG>CCT  GCA>GCC | None  None | T2N0M0 | III/III | N | N |
| FBC2 | 62^1^  175 | GCA>GCC  CCC>CTC | None  Pro>Leu | T2N1M0 | III/III | P | P |
| FBC3 | None | None | None | T2N0M0 | III/III | N | NA |
| FBC4 | None | None | None | T2N0M0 | III/III | N | P |
| FBC5 | None | None | None | T1N0M0 | II/III | N | N |
| FBC6 | 62^1^  175 | GCA>GCC  CCC>CTC | None  Pro>Leu | T2N1M0 | III/III | P | P |
| FBC7 | None | None | None | T3N1M0 | II/III | P | P |
| FBC8 | None | None | None | T3N0M0 | III/III | N | P |
| FBC9 | None | None | None | T1N0M0 | III/III | N | P |
| FBC10 | None | None | None | T1N0M0 | III/III | N | N |
| FBC11 | none | None | None | T1N0M0 | III/III | N | P |
| FBC12 | 175 | CCC>CTC | Pro>Leu | T1N1M0 | III/III | P | P |
| FBC13 | None | None | None | T2N0M0 | III/III | N | P |
| FBC14 | None | None | None | NA | NA | N | P |
| FBC15 | None | None | None | T1N0M0 | III/III | N | N |
| FBC16 | None | none | None | T2N1M0 | III/III | P | P |
| FBC17 | None | None | None | T3N1M0 | II/III | P | N |
| FBC18 | 175 | CCC>CTC | Pro>Leu | T1N1M0 | III/III | P | P |
| FBC19 | None | None | None | NA | NA | N | N |
| FBC20 | None | None | None | T2N0M0 | III/III | N | NA |
| FBC21 | None | None | None | T1N0M0 | III/III | N | N |
| FBC22 | none | None | None | T1N0M0 | III/III | N | P |
| FBC23 | None | None | None | T1N0M0 | III/III | N | N |
| FBC24 | None | None | None | T1N0M0 | III/III | N | N |
| FBC25 | 62^1^  175 | GCA>GCC  CCC>CTC | None  Pro>Leu | T2N1M0 | III/III | P | P |
| FBC26 | None | None | None | T1N0M0 | III/III | N | N |
| FBC27 | none | None | None | T1N0M0 | III/III | N | NA |
| FBC28 | None | None | None | T1N0M0 | III/III | N | N |

LOH, loss of heterozygosity; P, positive; N, negative; NA, not available; ^1^SNP, single nucleotide polymorphism
