# Supplementary material for: Clinical Implications of Rabphillin-3A-Like Gene Alterations in Breast Cancer
Source: PLoS One. 2015 Jun 12;10(6):e0129216. doi: 10.1371/journal.pone.0129216 (PMC4466565; doi:10.1371/journal.pone.0129216)
Supplement: S2 Table — (DOCX) [file pone.0129216.s003.docx]

**S2 Table:** Relationship between clinicopathological features and the combinations of *RPH3AL* and *TP53* LOH status in breast cancers.

| **Characteristic^1^** | ***RPH3AL* LOH +ve and *TP53* LOH +ve** | ***RPH3AL* LOH +ve and  *TP53* LOH -ve** | ***RPH3AL* LOH -ve and *TP53* LOH +ve** | ***RPH3AL* LOH -ve and *TP53* LOH -ve** | **P-value** |
| --- | --- | --- | --- | --- | --- |
| **Age Group (years)** |  |  |  |  |  |
| ≤ 50 | 15 (43) | 7 (19) | 7 (19) | 7 (19) | 0.612 |
| > 50 | 37 (54) | 12 (17) | 12 (17) | 8 (12) |  |
| **Ethnicity** |  |  |  |  |  |
| Caucasian | 29 (45) | 12 (19) | 13 (20) | 10 (16) | 0.792 |
| African-American | 22 (55) | 7 (18) | 6 (15) | 5 (12) |  |
| **Tumor size** |  |  |  |  |  |
| ≤ 2 cm | 18 (44) | 4 (10) | 9 (22) | 10 (24) | **0.042^2^** |
| > 2 cm | 34 (53) | 15 (23) | 10 (16) | 5 (8) |  |
| **Histologic grade** |  |  |  |  |  |
| I | 5 (38) | 2 (15) | 5 (38) | 1 (8) | 0.372 |
| II | 10 (46) | 4 (18) | 4 (18) | 4 (18) |  |
| III | 28 (62) | 3 (7) | 8 (18) | 6 (13) |  |
| **ER/PR status** |  |  |  |  |  |
| Positive | 27 (42) | 11 (17) | 16 (25) | 10 (16) | 0.169 |
| Negative | 21 (60) | 7 (20) | 3 (9) | 4 (11) |  |
| **Nodal status** |  |  |  |  |  |
| Positive | 25 (57) | 9 (21) | 5 (11) | 5 (11) | 0.235 |
| Negative | 21 (41) | 9 (17) | 12 (23) | 10 (19) |  |
| **Tumor Stage** |  |  |  |  |  |
| I | 10 (32) | 6 (19) | 8 (26) | 7 (23) | 0.136 |
| II | 24 (56) | 6 (14) | 8 (19) | 5 (11) |  |
| III&IV | 15 (60) | 6 (24) | 1 (4) | 3 (12) |  |

^1^Not all patients had information available for all the clinicopathological features; ^2^Fisher exact
